# Non-invasive Urinary Biomarkers in Moyamoya Disease

**DOI:** 10.3389/fneur.2021.661952

**Published:** 2021-04-01

**Authors:** Julie Sesen, Jessica Driscoll, Alexander Moses-Gardner, Darren B. Orbach, David Zurakowski, Edward R. Smith

**Affiliations:** ^1^Vascular Biology Program, Boston Children's Hospital and Harvard Medical School, Boston, MA, United States; ^2^Department of Neurosurgery, Boston Children's Hospital and Harvard Medical School, Boston, MA, United States; ^3^Department of Radiology, Boston Children's Hospital and Harvard Medical School, Boston, MA, United States; ^4^Departments of Surgery and Anesthesiology, Boston Children's Hospital and Harvard Medical School, Boston, MA, United States

**Keywords:** pediatric, biomarker, urine, cerebrospinal fluid, non-invasive, stroke, angiogenensis, moyamoya

## Abstract

**Introduction:** A major difficulty in treating moyamoya disease is the lack of effective methods to detect novel or progressive disease prior to the onset of disabling stroke. More importantly, a tool to better stratify operative candidates and quantify response to therapy could substantively complement existing methods. Here, we present proof-of-principle data supporting the use of urinary biomarkers as diagnostic adjuncts in pediatric moyamoya patients.

**Methods:** Urine and cerebrospinal fluid specimens were collected from pediatric patients with moyamoya disease and a cohort of age and sex-matched control patients. Clinical and radiographic data were paired with measurements of a previously validated panel of angiogenic proteins quantified by ELISA. Results were compared to age and sex-matched controls and subjected to statistical analyses.

**Results:** Evaluation of a specific panel of urinary and cerebrospinal fluid biomarkers by ELISA demonstrated significant elevations of angiogenic proteins in samples from moyamoya patients compared to matched controls. ROC curves for individual urinary biomarkers, including MMP-2, MMP-9, MMP-9/NGAL, and VEGF, showed excellent discrimination. The optimal urinary biomarker was MMP-2, providing a sensitivity of 88%, specificity of 100%, and overall accuracy of 91%. Biomarker levels changed in response to therapy and correlated with radiographic evidence of revascularization.

**Conclusions:** We report, for the first time, identification of a panel of urinary biomarkers that predicts the presence of moyamoya disease. These biomarkers correlate with presence of disease and can be tracked from the central nervous system to urine. These data support the hypothesis that urinary proteins are useful predictors of the presence of moyamoya disease and may provide a basis for a novel, non-invasive method to identify new disease and monitor known patients following treatment.

## Introduction

Moyamoya disease is an increasingly recognized cause of pediatric stroke, found in ~6% of cases in the United States (1, 2). Surgical revascularization is an effective treatment, but clinicians face challenges in successfully identifying disease prior to disabling stroke, predicting optimal timing for operative intervention and tailoring specific surgical approaches to a given patient. Follow-up is important to ensure successful engraftment and to monitor for progressive disease in other vascular territories. These challenges are further exacerbated in children, who may not be able to articulate symptoms clearly or tolerate detailed imaging more easily and safely performed in adults.

Our laboratory has had a longstanding interest in the development of non-invasive biomarkers designed to aid in the diagnosis, prognosis and therapy of tumors and cerebrovascular disease, including biomarker “fingerprints” that can distinguish between central nervous system tumors, moyamoya disease and arteriovenous malformations (3–10). Here we present initial proof-of-principle data demonstrating that a novel, non-invasive panel of urinary biomarkers can identify the presence of moyamoya disease and that the biomarkers track from the central nervous system in cerebrospinal fluid (CSF) to urine. Importantly, we also show that levels of these biomarkers vary in response to therapeutic intervention and correlate with radiographic changes post-surgery. To our knowledge, this is the first report of this application of urinary biomarkers in this population and we hope that these data provide a foundation for expanded study of this approach.

## Materials and Methods

### Patient Population

Specimens and records were collected as part of an institutional review board-approved protocol at Boston Children's Hospital (BCH) and patients underwent sample collection and surgery between 2009 and 2016. All moyamoya disease patients were 18 years of age or younger at time of specimen collection and all moyamoya disease diagnoses were confirmed with MRI and catheter angiography with independent verification by board-certified neuroradiologists as part of routine clinical practice and documented in the medical records. To standardize patient populations as much as possible, all patients were pediatric, met the diagnosis of moyamoya disease (not syndromic) with bilateral disease, Suzuki grade II-V and at least 6 weeks out from any documented acute stroke or hemorrhage at time of collection to minimize the risk of confounding biomarker profiles from acute stroke (11, 12). This timeline was based on previous data that indicates that any alterations in the levels of the biomarkers examined in this study—including MMPs and VEGF—that could potentially be affected by acute stroke are typically normalized within this time window (11, 13–15). No patients had known histories of other vascular malformations or recent surgery (within 3 months of specimen collection).

Control patients were healthy, age- and sex-matched and had undergone previous unremarkable imaging of the head and brain as part of routine clinical care (typically negative studies following evaluations to rule out congenital pathologies such as Chiari I malformation or tethered spinal cord).

### Urine and CSF Collection and Analysis

Urine from moyamoya disease patients was collected in the morning before surgery (or at the 6–12 month postoperative visit) and CSF was collected at time of surgery from the craniotomy site. Urine and CSF of control patients was collected from children undergoing operation for simple fatty filum/tethered cord or collected as part of routine clinical care. Once collected, urine and CSF were transported on ice to our laboratory and stored frozen (−20°C) until assay. Aliquots of each sample were centrifuged at 4,000 rpm for 5 min at 4°C and the supernatants were collected, as previously described by us (4).

ELISA (Quantikine kits; R&D Systems, Inc.) were used to quantify levels of MMP-2, MMP-9, MMP-9/NGAL, and VEGF. Specimens, standards and reagents were prepared according to manufacturer's instructions. Protein concentration was determined via the Bradford method using bovine serum albumin as the standard. Levels were determined as nanogram per milliliter (ng/mL) for MMP-2, MMP-9, and MMP-9/neutrophil gelatinase—associated lipocalin (NGAL) or picogram per liter (pg/L) for VEGF.

This work is presented in accordance with reporting recommendations for biomarker prognostic studies (16).

### Statistical Analysis

Statistical analysis was performed by our biostatistician (DZ). Patients with moyamoya disease and age-matched controls were compared with respect to urinary MMPs (ng/mL) and VEGF (pg/L) by the univariate Mann–Whitney *U*-test and presented using medians and interquartile ranges (17). Percentages of individuals positive for MMP-9, MMP-9/NGAL, MMP-2, and VEGF were compared between the two groups using Fisher's exact test for binomial proportions. Receiver operating characteristic (ROC) curve analysis was performed to calculate area under the curve (AUC) for the independent predictors and to identify threshold values (i.e., cut-off points) that provide the optimal tradeoff between sensitivity of specificity (18). AUC values and 95% confidence intervals (CI) were used to summarize diagnostic performance of MMPs and VEGF.

The AUC value (maximum 1, also known as the c-index) was used as a measure of predictive accuracy of each biomarker. Multivariable logistic regression modeling was applied to determine significant independent predictive biomarkers of moyamoya disease (19). Statistical analysis was conducted using SPSS software version 24.0 (IBM Corporation, Armon, NY). Power analysis indicated that the sample sizes of moyamoya disease patients and age-matched controls provided 80% power (α = 0.05, β = 0.20) to detect differences of 50% in the proportion of individuals with positive expression (20). Two-tailed *P* < 0.05 were considered statistically significant. AUC values over 0.700 were considered good and values over 0.800 were regarded to indicate excellent predictive accuracy.

## Results

### Demographics

A total of 32 patients with moyamoya disease (17 females, 15 males) and 14 healthy controls (7 females, 7 males) from 1 to 18 years of age were included in this study. Median age (interquartile range) of patients and controls were 8 (7–14) years and 7 (4–13) years, respectively (*P* = 0.20). Gender distribution did not differ between the two groups analyzed for urinary biomarkers (*P* = 0.85) (Table 1). The CSF subset of this cohort was smaller (*n* = 19 patients, *n* = 5 controls) and were also age and sex-matched (*P* = 0.49 and 0.90, respectively). Of the 32 moyamoya disease patients, 22 had experienced previous radiographic stroke (69%) and 26 (81%) had experienced transient ischemic attacks (TIAs), but no patients had new strokes within 6 weeks of sample collection (as documented by diffusion weighted imaging on MRI).

**Table 1:** Comparison of urinary MMPs and VEGF for moyamoya disease patients and controls.

	**Moyamoya group (*****n*** **= 32)**			**Control group (*****n*** **= 14)**			
**Biomarker**	**Median**	**IQR**	**Range**	**Median**	**IQR**	**Range**	***P-*value**
MMP-2	11.7	3–19.6	0–91	0	0–0	0–0	<0.001^*^
MMP-9	0.2	0–9.7	0–273	0	0–0	0–6.2	0.005^*^
MMP-9/NGAL	1.0	0.4–5.3	0.2–149	0	0–0.1	0–12.8	<0.001^*^
VEGF	420	163–1,112	0–4,000	0	0–113	0–391	<0.001^*^

*Units are ng/mL for MMPs and pg/L for VEGF. IQR, interquartile range*.

*MMP-9/NGAL and VEGF are based on 20 moyamoya patients*.

* *Statistically significant*.

### Urinary Biomarkers Are Elevated in Moyamoya Disease Patients

**Figure 1:**
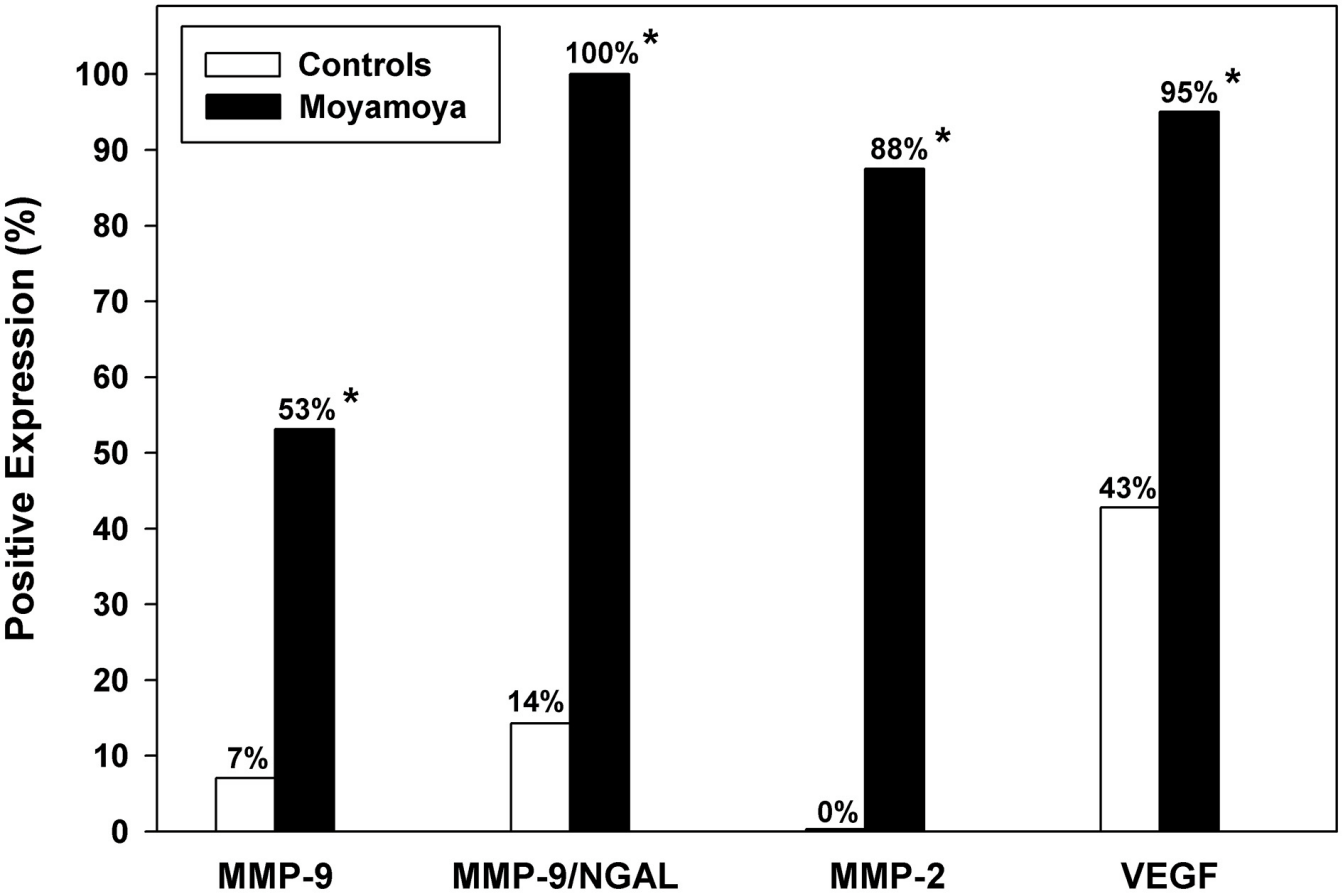
Comparison of the percentage of moyamoya disease patients and controls with positive expression for urinary MMPs and VEGF. Significantly higher positive expression rates were observed for each biomarker as denoted by asterisks. The most useful biomarker according to both multivariable logistic regression and ROC analysis was MMP-2 demonstrating excellent predictive accuracy.

Median levels of urinary MMPs and VEGF were significantly higher among moyamoya disease patients (all *P* < 0.001, except MMP-9, *P* = 0.005, Mann–Whitney *U*-tests). As shown in Table 1, median levels of MMP-2 were 11.7 ng/mL (IQR: 3.0–19.6) for patients and 0 ng/mL (IQR: 0–0) for controls. Median VEGF levels were 420 pg/L (IQR: 163–1,112) and 0 pg/L (IQR: 0–113) for patients and controls, respectively. As illustrated in Figure 1, a significantly higher percentage of moyamoya disease patients (17 of 32 = 53%) were positive for MMP-9 compared to controls (1/14 = 7%) (*P* = 0.003). Similarly, whereas only 2 of 14 controls were positive for MMP-9/NGAL, all 20 of the moyamoya disease patients tested were positive (*P* < 0.001). A total of 88% of moyamoya disease patients were positive for MMP-2 (28 of 32) compared to 0 of 14 controls (*P* < 0.001). For VEGF, 95% of patients (19 of 20) were positive compared to 43% (6 of 14) controls (*P* < 0.01).

**Figure 2:**
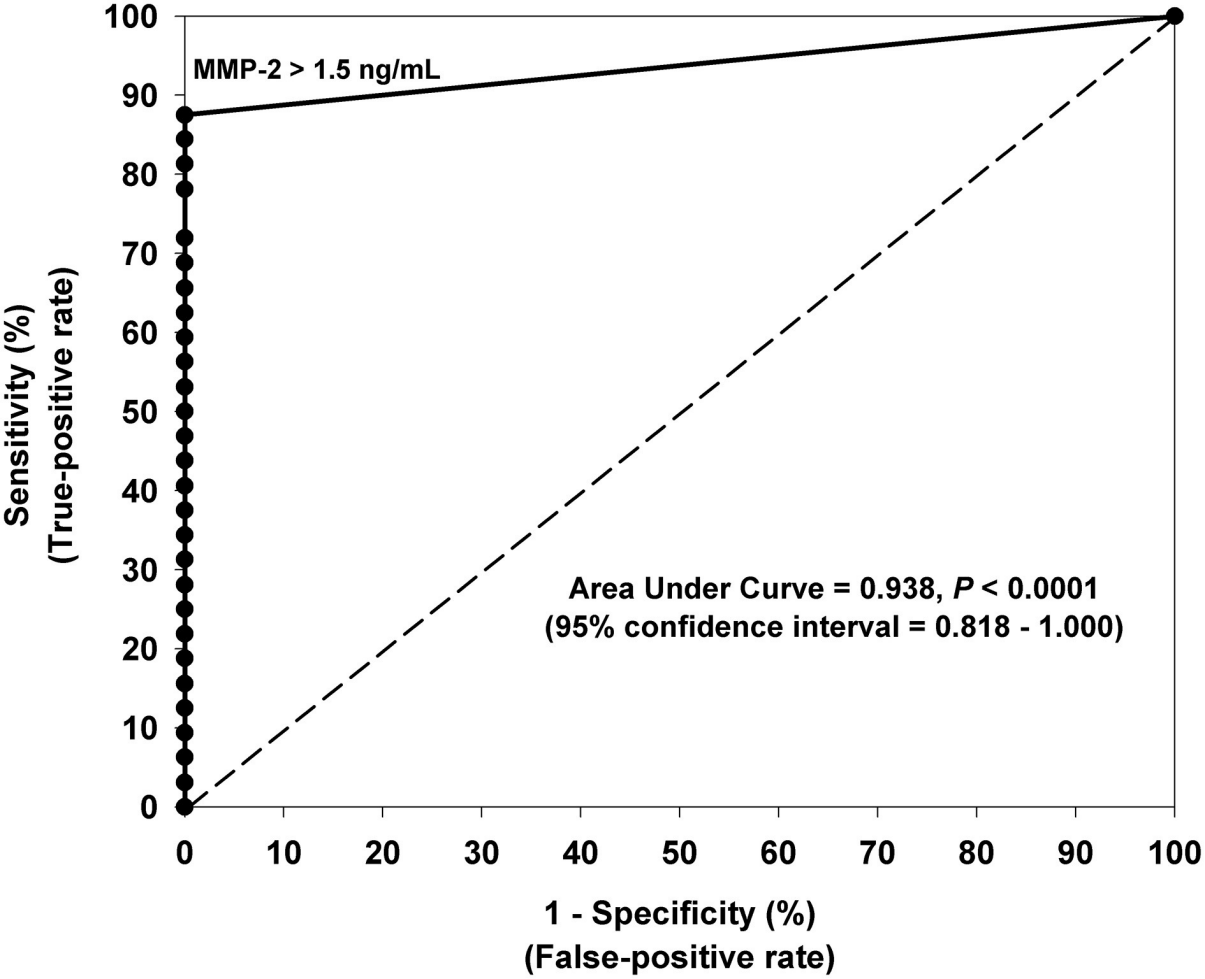
Receiver-operating characteristic (ROC) curve analysis indicating the optimal urinary MMP-2 cut-off value (>1.5 ng/mL) for differentiating moyamoya disease patients and controls. The 45° line represents the line of nondiscrimination which would be equivalent to a coin toss. The area under the ROC curve for MMP-2 indicates excellent diagnostic predictive accuracy (AUC = 0.938, 95% CI: 0.870–1.000, *P* < 0.0001). Sensitivity was 88% and specificity was 100% using the optimal cut-off value of 1.5 ng/mL.

Receiver-operating characteristic (ROC) curve analysis indicated that all urinary MMPs and VEGF were significant in differentiating moyamoya disease patients from healthy controls as indicated by the area under the curve (AUC) values (Table 2). AUC for MMP-2, indicating diagnostic performance, was excellent (AUC = 0.938, 95% CI: 0.870–1.000, *P* < 0.001). ROC analysis revealed the optimal cut-off value for MMP-2 as 1.5 ng/mL, which is associated with a sensitivity of 0.88 or 88% (28 of 32 moyamoya disease patients correctly classified), a specificity of 1.00 or 100% (all 14 controls correctly classified), and an accuracy of 91% (42/46). Any non-zero cut-off value or criterion for MMP-2 would have produced false negatives since four moyamoya disease patients did not have measurable MMP-2 in their urine. Figure 2 depicts the ROC curve and shows the relationship between the true-positive rate and the false-positive rate with the optimal urinary MMP-2 cut point of >1.5 ng/mL.

**Table 2:** ROC Analysis of urinary biomarkers in predicting moyamoya disease.

**Biomarker**	**AUC**	**95%** **Confidence Interval**	***P*-value**
MMP-2	0.938	0.870–1.000	<0.001^*^
MMP-9	0.731	0.587–0.877	0.013^*^
MMP-9/NGAL	0.933	0.818–1.000	<0.001^*^
VEGF	0.875	0.756–0.994	<0.001^*^

*ROC, receiver operating characteristic; AUC, area under the curve*.

* *Statistically significant*.

Multiple stepwise logistic regression revealed that, independent of age and gender, urinary MMP-2 was the only independent biomarker that significantly differentiates moyamoya disease patients from controls (c-index = 0.938, *P* < 0.001). Although MMP-9 and MMP-9/NGAL also demonstrate significant diagnostic performance, as indicated by the statistics in Table 2, the results of the ROC analysis and logistic regression modeling clearly showed that MMP-2 prevailed as the most accurate urinary biomarker in differentiating moyamoya disease from normal healthy controls.

### CSF Biomarkers Are Elevated in Moyamoya Disease Patients

CSF analysis was carried out in a similar fashion to that done on the urine and was collected from the same individuals. CSF analysis was constrained by both the number of patients available for the study and the volume of useable CSF per patient (due to fewer controls and the limitations in getting adequate volumes of non-bloody CSF for collection per individual, both controls and moyamoya disease). These limitations meant that we had a smaller “*n*” of patients, and the volume of CSF per patient meant that we had to limit our CSF biomarker analysis. Consequently, we focused on the MMP species identified as most promising from the urinary studies and were forced to exclude VEGF from CSF analysis.

We applied ROC analysis to evaluate the diagnostic performance of MMPs in CSF (Table 3). Of the three MMPs studied, only MMP-9 was predictive (AUC = 1.000, *P* = 0.002), although MMP-2 and MMP-9/NGAL also had strong trends toward significance. The optimal cut-off value for MMP-9 in CSF was 2.6 ng/mL which was associated with a sensitivity and specificity of 100%. All 5 controls had MMP-9 levels <2.6 ng/mL (three had no detectable levels of MMP-9, one was 0.2 ng/mL, and one subject had a value of 2.2 ng/mL). Of the 19 moyamoya disease patients with MMP-9 measurements in CSF, levels in all patients ranged from 2.9 to 27.0 ng/mL. Multiple stepwise logistic regression confirmed that, controlling for age and gender, MMP-9 was independently predictive of moyamoya disease (*P* = 0.003).

**Table 3:** ROC analysis of markers in cerebral spinal fluid for differentiating patients with moyamoya disease from controls.

**CSF marker**	**AUC**	**95% CI**	***P*-value**
MMP-2	0.274	0.000–0.600	0.126
MMP-9	1.000	1.000–1.000	0.002^*^
MMP-9/NGAL	0.776	0.569–0.984	0.066

*ROC, receiver operating characteristic; AUC, area under the curve; CI, confidence interval*.

* *Significant multivariable predictor*.

*Optimal cut-off value for MMP-9 is 2.6 ng/mL or greater. This cut-off value provides a sensitivity of 100%, specificity of 100%, and accuracy of 100%*.

### Urinary Biomarkers Change and Correlate With Postoperative Radiographic Response

**Figure 3:**
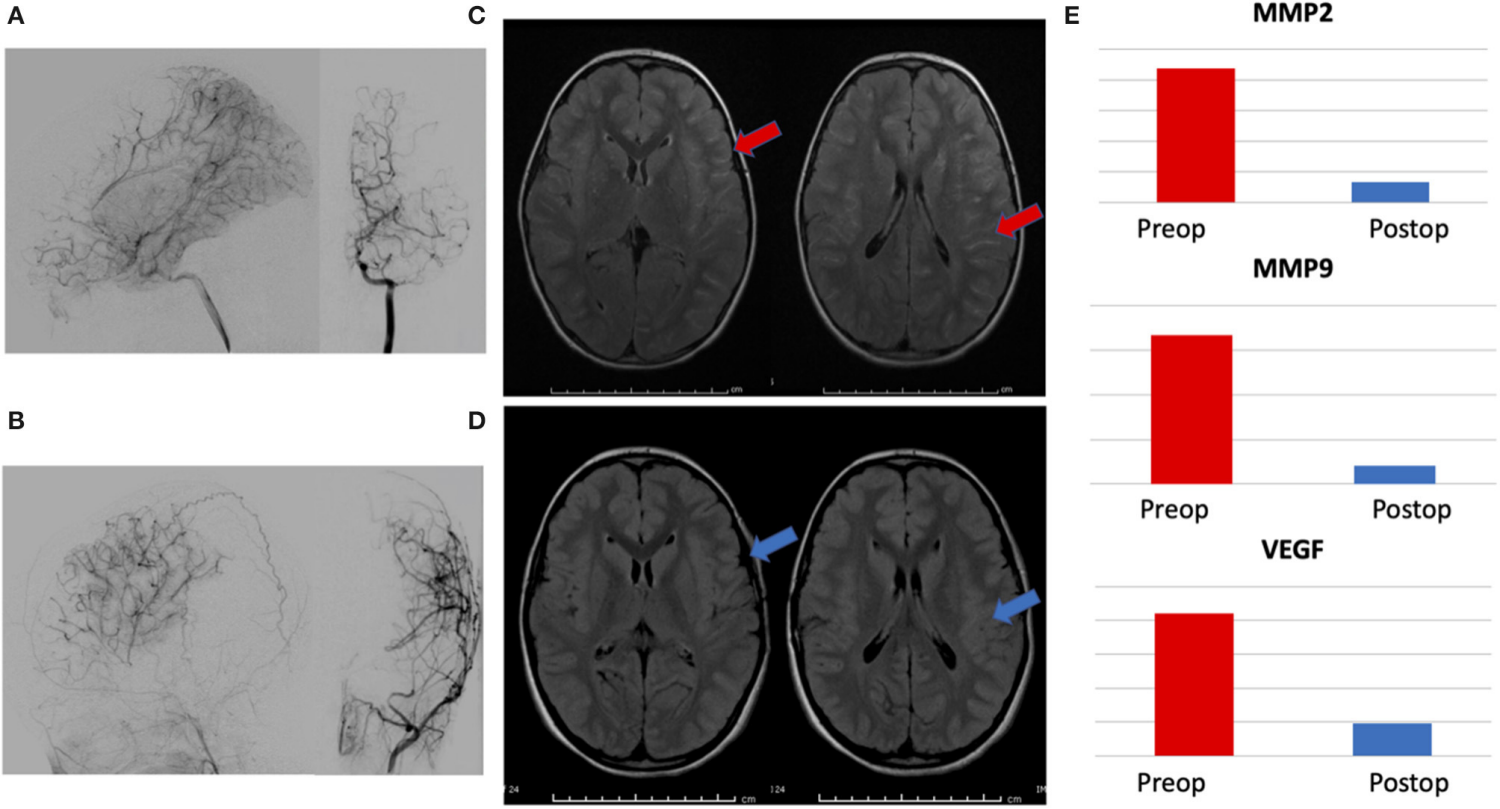
Representative patient demonstrating correlation between radiographic changes and biomarker levels. **(A)** Preoperative angiogram with lateral and AP views of internal carotid artery injection showing Suzuki II-III moyamoya, **(B)** 1 year postoperative angiogram with lateral and AP views of external carotid injection showing Matsushima A surgical collaterals, **(C)** preoperative axial FLAIR MRI images demonstrating hyperintense sulcal signal—ivy sign, (red arrows) and **(D)** postoperative images showing marked resolution of ivy sign (blue arrows). **(E)** Reduction in postoperative levels of urinary biomarkers MMP-2, MMP-9, and VEGF, correlating with radiographic evidence of successful revascularization.

Urine was collected at 6–12 months postoperatively in patients within this group. A total of 7/32 patients had urine available for analysis. Biomarker levels were compared to preoperative samples (MMP-9/NGAL ELISA kits were no longer unavailable at the time of this delayed analysis, so MMP-2, MMP-9, and VEGF were performed). Pre- and post-operative angiograms were assessed for surgical collaterals (as measured by Matsushima grade) and pre- and post-operative axial FLAIR MRI images were compared for changes in ivy sign (a marker of slow cortical blood flow). All patients had Matsushima A or B collaterals and had reductions in global ivy sign as independently read by neuroradiologists as part of routine clinical care. MMP-2 levels decreased in 6/7 (86%) patients, MMP-9 and VEGF decreased in 5/7 (71%) patients. Representative data from a patient is presented in Figure 3.

## Discussion

### Rationale for Urinary Biomarkers: *Why urine?*

There is ample precedence for the successful use of urinary biomarkers to identify physiologic states such as pregnancy and to monitor disease, such as diabetes. Use of urinary biomarkers offers advantages particularly relevant to moyamoya disease. Current methods of diagnosis and follow-up rely on infrequent clinical examinations and expensive radiographic studies (such as MRI and angiograms) that also often require sedation or anesthesia in children (2, 21). In contrast, urine collection can be done frequently, without sedation and is relatively inexpensive. Collection of urine is easy and non-invasive, avoiding the difficulties and risk inherent to biomarkers studied in spinal fluid or blood, which require lumbar punctures and venipuncture. Urine collection can be done locally and mailed, saving families travel to hospitals (10). Most importantly, biomarkers provide a method of diagnosis that relies on biological activity; a novel, quantifiable—and complementary—approach to the current method of evaluation with imaging studies.

### Selection of Moyamoya Disease Biomarker Panel: *Why choose these proteins?*

#### Background

Our laboratory has extensive experience with the identification and validation of urinary biomarkers in cerebrovascular disease, cancer and the central nervous system (CNS), including the first report of successfully applying this novel methodology specifically to brain tumors in a multicenter trial (3–10). Current data from our lab and others supports the hypothesis of angiogenesis and extracellular matrix (ECM) remodeling as essential processes in many CNS disorders, including moyamoya disease, involving vascular endothelial growth factor (VEGF), matrix metalloproteinases (MMPs), a multigene family of degradative enzymes and neutrophil gelatinase associated lipocalin (NGAL), an enzyme that binds with MMP-9 to protect it from degradation—and elevated levels of MMPs and VEGF have been reported in the serum of moyamoya disease patients (2, 4, 5, 22–30). Our group was the first to demonstrate that human urinary MMP levels are sensitive biomarkers of various systemic disorders, including cancer and vascular anomalies (4, 5, 21, 31–40). Recent studies from our laboratory, now confirmed by others, support the utility of urinary levels of growth factors and MMPs as markers of disease, including our work first describing the use of this technique specifically in the CNS (4, 5, 11, 21, 26, 37, 40–48).

#### Rationale

In the study presented here, we focused on MMPs, NGAL, and VEGF as proteins known to be involved in the pathogenesis of moyamoya disease and easily detectable in urine with commercially available methods (in contrast to more complex techniques, such as mass spectrometry). It is important to note that these are useful as general markers of ischemia/ECM remodeling—and therefore potentially directly applicable to monitoring clinical factors relevant to moyamoya disease status and progression—but are not unique to this condition. In addition, some markers—like the ones in this study—may be elevated immediately after stroke, but typically normalize after a short window of a few weeks, making the timing of biomarker sampling important and which we have addressed in the selection of our patient cohort to minimize confounding (11, 13–15). Diagnostic specificity can be improved by looking at a broader range of molecules and multiplexing several proteins in combination to create a biomarker “fingerprint” that is unique to a given disease, and we have done this for other CNS vascular and tumor cohorts, including moyamoya disease (3–5, 10, 49). In particular, we anticipate that molecules that regulate mechanisms of vascular morphogenesis and arteriogenesis (including, but not limited to, axon guidance factors, for example), may be useful as future areas of investigation to enhance the functionality of biomarker profiling in moyamoya disease.

#### Correlation of Urine to CNS Disease

Another key point of this study is the ability to link the levels of these putative biomarkers with the disease. A unique problem with moyamoya disease is the inability to harvest source tissue for analysis, distinct from other pathologies such as tumor and AVM, that afford primary lesional tissue for confirmation with biomarker staining. We addressed this challenge with three strategies. First, we subjected the urinary data to rigorous statistical analysis, performed by a biostatistician, in order to ensure the robustness of our results. Second, we obtained matched CSF samples from the same patients, in order to establish that the biomarker profile in the CNS matched the same pattern seen in the urine, a technique validated in the literature and—to our knowledge—unique in its application to moyamoya disease as described here (3–5). Third, we provide longitudinal data that demonstrates that the urinary biomarker profile changes in direct response to changes in disease status after treatment, as corroborated by radiographic studies and also with precedent in the literature (3–6, 10, 49).

### Potential Applications to Moyamoya Disease: *How might this approach be used?*

Currently there are no methods available that can accurately predict how a given child's disease will progress or how that child may respond to surgical intervention (2). While the precedent of prognostic biomarkers has proven immensely valuable in other fields of medicine to predict outcomes or response to therapy, nothing like this exists for moyamoya disease (50–52). While in need of validation, the diagnostic potential of this panel could be expanded to incorporate a prognostic function by correlating high pre-operative levels of biomarker with good potential for revascularization and concomitant reduction in post-operative stroke risk. Development of a prognostic biomarker panel as outlined in this proposal would bring something entirely new—and needed—to the armamentarium of clinicians treating patients with moyamoya disease.

This study fulfills the necessary initial criteria in clinical biomarker development; statistical validation of a panel of markers that can be accurately measured with widely available techniques and associated with a biologically relevant pathway that connects the biomarkers to the disease process (9, 16). In addition, this work is unique in linking these markers from CSF to urine and also presenting their utility in a “real-world” clinical scenario of longitudinal follow-up. Ultimately, we would anticipate a panel of biomarkers for moyamoya disease (not a single protein), with distinct combinations employed based on the clinical need. For example, one fingerprint might be applied for screening, while a different group of biomarkers might help to stratify ischemia and follow response to surgery.

There is precedent to use biomarkers as tools to monitor response to therapy. Specifically, in moyamoya disease and other vascular diseases, evidence exists that levels of MMPs and VEGF are elevated in the setting of chronic ischemia and—once successful revascularization occurs and the ischemia is reduced—the ischemic stimulus driving the upregulation of MMP and VEGF is decreased, with a concomitant reduction in the levels of these markers (27, 30, 49, 53–55). As documented in previous work from our lab and others, the CNS levels of these molecules are directly related to urinary levels, with previous reports linking source tissue, CSF, serum and urine (3–6, 21, 37, 56). The working model is that the end-organ (brain) experiences chronic ischemia from the moyamoya arteriopathy and is elaborating these angiogenic factors at baseline in order to develop compensatory collateral development. Once surgical revascularization occurs, transient elevations in these factors enhance surgical collateral growth until the ischemia is corrected (a process well-documented by postoperative imaging studies, showing improved perfusion and surgical collaterals on angiogram), at which point the ischemic stimulus no longer exists, and the production of pro-angiogenic molecules decreases. While we do not have sampling from the immediate postoperative period (days to weeks), we would hypothesize that we would see marked elevations in pro-angiogenic biomarkers (such as VEGF and MMPs) within this window until enough revascularization occurred to normalize perfusion (usually several weeks to months after indirect revascularization). In support of this hypothesis, our data shows that these biomarkers are elevated pre-surgery and decrease post-operatively in concordance with radiographic evidence of effective surgical revascularization on angiogram and improved hemodynamic perfusion on MRI with reduced ivy sign (Figure 3).

To be clear, the rarity of moyamoya disease suggests that widespread screening of the general population to identify *de novo* cases is not a realistic goal. Utility would be greater in screening a defined, at-risk population, such as Down syndrome (which has a 26-fold higher incidence of moyamoya syndrome), sickle cell disease or patients with known family histories of moyamoya (1, 2, 9, 57–59). This targeted approach could reduce risk of scanning, cost of screening and aid in better detection of disease. Another important role for biomarkers in moyamoya disease may be aiding in stratification of risk and operative strategy in already identified moyamoya patients, with biomarker-based risk reassessment on an ongoing basis. Longitudinal data from this study suggest that these biomarkers change in response to ischemia, and further work may allow clinicians to better select operative candidates by adding biomarker profiling to complement image analysis. This could be especially useful in the growing number of asymptomatic or early-stage moyamoya disease cases that are presently without a clear clinical equipoise.

Finally, the role of urinary biomarkers may extend beyond diagnostic or prognostic adjuncts and actually inform the development of novel, biologically-based therapies. This approach of combining a specific therapy with immediate feedback on efficacy—theranostics—has rapidly expanded in medicine. Our lab has started to merge the fields of diagnostic biomarkers with targeted therapeutics in brain tumors (60, 61). It is tempting to consider that a similar approach with moyamoya disease, using biomarker-informed delivery of pro-angiogenic therapeutics, then following response to therapy, as shown in this paper.

### Limitations and Future Directions

Although much of the data revealed by this work is promising, there are clearly a number of limitations inherent to this study. First, a challenge is the rarity of the disease, making it difficult to collect CSF and urine from the same patient in great numbers. For example, while only one of the CSF markers in this study reached significance, the others clearly trended as expected and we anticipate that validation would have been achieved with larger datasets. It will be important in future work to substantially increase the CSF sampling if it is to be as robust as urinary data. Also relevant to the rarity of patients is expanding the number of post-operative urine samples to validate this approach, as minimizing variation related to timing and recovery from surgery will be important. To address this issue, it would be ideal to validate these findings in much larger populations, ideally as multi-center effort. Second, there is potential heterogeneity in moyamoya, with both disease and syndromic populations (2). While we have tried to minimize this variability by standardizing age, disease status and linked specimens, it would be helpful to expand this study to examine defined cohorts of moyamoya (RNF213, Down syndrome, post-radiation, etc.) and determine if there are unique biomarker signatures across populations. Third, expanding the number of molecules to evaluate as putative biomarkers for specific clinical applications could be done with larger numbers—and this has been successfully achieved in multiple diseases, by our group and others. For example, a panel of biomarkers designed specifically to quantify ischemia could be developed in conjunction with radiographic data.

## Conclusions

We report a novel panel of urinary and CSF biomarkers that can identify the presence of moyamoya disease with a high degree of sensitivity and accuracy. Urinary MMP-2 emerged as the optimal marker, with a sensitivity of 87.5%, specificity of 100%, and accuracy of 91.3%. We demonstrate that these biomarkers track from CSF to urine and correlate with response to therapeutic interventions, including evidence of radiographic revascularization. These proteins can be assessed non-invasively, offering unique advantages in safety, ease of monitoring and reduced cost, along with a new quantifiable, biological approach that complements existing clinical and radiographic practice. Together, these proof-of-principle data support the ongoing investigation of urinary biomarkers as tools that may have utility in the diagnosis, prognosis, and treatment of moyamoya disease.

## Data Availability Statement

The datasets presented in this article are not readily available because they are linked to patient clinical images and histories and the assays are commercially available, as noted in the Methods section. Requests to access the datasets should be directed to edward.smith@childrens.harvard.edu.

## Ethics Statement

The studies involving human participants were reviewed and approved by Boston Children's Hospital Institutional Review Board. Written informed consent from the participants' legal guardian/next of kin was not required to participate in this study in accordance with the national legislation and the institutional requirements.

## Author Contributions

ES and JS contributed to conception and design of the study. JD and AM-G analyzed the data. DZ performed the statistical analysis. ES wrote the first draft of the manuscript. ES, DZ, and DO wrote sections of the manuscript. All authors contributed to manuscript revision, read, and approved the submitted version.

## Conflict of Interest

The authors declare that the research was conducted in the absence of any commercial or financial relationships that could be construed as a potential conflict of interest.
